# Book Reviews

**Published:** 1824-06

**Authors:** 


### CRITICAL ANALYSIS
### OF
### ENGLISH AND FOREIGN LITERATURE
### RELATIVE TO THE VARIOUS BRANCHES OF
### Medical Science.

Quæ laudanda forent, et quæ culpanda, vicissim

Illa, prius, cretâ; mox hæc, carbone, *notamus.*—PERSIUS.

### DIVISION I.

### ENGLISH.

ART. I.—*Observations on Injuries of the Spine and of the Thigh-bone, in two Lectures delivered in the School of Great Windmill-street. The first in vindication of the Author's Opinions against the Remarks of* Sir ASTLEY COOPER, Bart.; *the second on the late* Mr. JOHN BELL's *Title to certain Doctrines, now advanced by the same Gentleman. Illustrated with nine Plates.* By CHARLES BELL, Surgeon to the Middlesex Hospital.—4to. pp. 108. London, 1824.

THE controversies which are carried on from time to time among the cultivators of literature and science are frequently of use to the public; and, perhaps, there are occasions when a sort of apathy steals over the members of our profession, which requires the excitement of some stronger stimulus than is necessary in a state of vigorous and active health. Some circumstances lead us to suspect that the medical profession in England is bordering upon this state of indolence and lethargy; and we hail, not without some hopes of salutary effects, the agitation which students in surgery will receive from a contest, which has originated in matters particularly addressed to them, and on their opinion of which must, in some degree, depend the estimate they form concerning the efficacy and the value of the lessons they have been taught in two of the most celebrated schools in this metropolis, the Borough and Great Windmill-street. In the heat of a contest which at one time shook the anatomical world, it was well observed, that "perhaps it may be as sound philosophy to say that all the actions of men are directed to some good end, as it is to subscribe to an opinion, which has prevailed among naturalists, that in the works of nature nothing is absolutely without its use. Literary disputes are disagreeable to the greatest part of mankind, and the disputants are for the most part condemned by the world; yet it is reasonable to think that even these disputes answer some good purpose. By engaging the passions of men more warmly, they rouse a spirit of emulation, and give a spur to inquiry.

"It is remarkable that there is scarce a considerable character in anatomy that is not connected with some warm controversy. Anatomists have ever been engaged in contention: and, indeed, if a man has not such a degree of enthusiasm and love of the art as will make him impatient of unreasonable opposition, and of encroachments upon his discoveries and his reputation, he will hardly become considerable in anatomy, or in any other branch of natural knowledge.

"These reflections afford some comfort to me, who unfortunately have been already engaged in two public disputes. I have imitated some of the greatest characters in what is commonly reckoned their worst parts; but I have also endeavoured to be useful, to improve and diffuse the knowledge of anatomy; and surely it will be allowed here, that, if I have not been serviceable to the public in this way, it has not been for want of diligence or love of the service."

Such are the sentiments which were expressed by the illustrious Dr. William Hunter, in his celebrated examination of the pretensions advanced by Mr. Pott to the discovery of the nature of congenital hernia; and we have been induced to quote them as applicable to the present work, which, as its title implies, is of a controversial nature. The most unpleasant circumstance attending discussions of this kind, is the manner in which disciples of the different parties step forward to vindicate their masters, and thus becoming principals in the contest, amid the heat of invective and dispute, too often misapprehend or overlook those points on which alone the general interest depends. In the present instance there is little risk of this secondary, and generally more unpleasant, discussion, Sir Astley Cooper and Mr. Charles Bell being fairly pitted against each other; the former having promulgated, in his great work on Dislocations, certain doctrines, some of which the latter claims as his brother's and his own; while he deprecates others as theoretically and practically erroneous.

Before we enter upon an analysis of Mr. Bell's work, we have to remark that this controversy seems to have been excited by the reports of Sir A. Cooper's lectures, which have appeared in some of the weekly journals, although his remarks on that gentleman's opinions are, with one exception, confined to his published and acknowledged writings.

"I do not feel at liberty," says Mr. Bell, "to quote the weekly journals of Sir Astley Cooper's lectures, as conveying his sentiments; but I think I am authorised to say, that, on reading what is there delivered, it was a duty which he owed to the profession, and to all his friends, to disavow, in the most public manner, expressions and words ascribed to him in these publications. This should have been done, even if handbills had been necessary, at the door of every class-room in London. Now it has happened that my pupils are sitting before me with these pamphlets in their hands. They are extensively circulated, and the question comes to be, how are the sentiments and opinions expressed there to be contradicted? For, that they require contradiction, I shall have no difficulty in convincing the profession." (P. viii.)

This is strong language; and we are rather astonished to find it coming from Mr. Bell, who has looked in silence on the apathy with which his discoveries in the nervous system have been received by many of his brethren, and left unanswered the gross plagiarism of the French physiologists, who, seeing the ingenuity and importance of his views, have surreptitiously adopted them as their own. We say the language in which Mr. Bell censures the expressions attributed to Sir A. Cooper is strong; but we do not think it too strong, if these expressions be correctly reported in the journals referred to; and, if they be not so, why have they not been refuted? Had the present discussion been one of a personal nature merely, and relating only to contending claims for priority of discovery, we should probably have taken no notice of it: but this is not the case, the work before us containing many very important facts, both in pathology and practice; and, if the collision of two such individuals should give rise to some heat, still we think surgery is likely to benefit by the discussion, although somewhat warm; while we, as unbiassed spectators, keep in mind that the main argument cannot be affected by quips and repartees, but by facts and reasoning. It is neither our business nor intention to take any part in this controversy, but rather to hold the balance between the parties with an impartial hand; such as we persuade ourselves we lately did in examining the disputed points between Sir A. Cooper and Mr. H. Earle.

We now proceed, without further preface, to lay before our readers an analysis of the principal opinions, which it is the object of the author to establish; in doing which, the remarks we have made above, will explain our motives for declining to make any comments.

The work consists of two Lectures,—the first on the Spine, "in which the accidents to which it is liable are explained, from the observation of the natural forms and connexions of the vertebræ;" the second on the Thigh-bone, "showing the applications of anatomy to practice." Mr. Bell commences his first Lecture by showing how apt young men are to form erroneous ideas regarding injuries of the head and of the spine, from the manner in which the anatomy of these parts is too frequently studied; and goes on to remark how soon a surgeon of discernment perceives that all other circumstances become unimportant, when compared to the state of the brain and its membranes; for on the condition of these it is that the question of life and death depends. "The mistaken and cruel practice" which formerly prevailed, being attributed to the attention having been more directed to the anatomy of the bones than to the structure and functions of the brain itself: and now, continues our author, "by a false analogy, and a course of most unsatisfactory and inconsequent reasoning, we are about to run into the same course of error in respect to injuries of the spine." Having spoken of the mechanism of the vertebral column, we find the following remarks on the soft parts connected with it.

"I now show you the spine, with the soft parts attached; the spinal marrow with its sheath; the ligaments and intervertebral substance. It is now that you are to receive that impression which will carry you through practice, and which is to distinguish you from the mere mechanist or instrument-maker. You see here the most vital organ enclosed within the tube of the spine, and surrounded with membranes the most prone to inflammation of any in the whole body. Here, then, is the consideration which should suspend your hand when you are about to put some ingenious mechanical notion into practice, which you expect to distinguish you in your profession: unless you have impressed on you a lasting conviction of the importance of these membranes, your ingenuity will prove a misfortune to some unhappy being: you may escape detection, but not your own lasting regret."

Mr. Bell next proceeds to inquire into the effects of the different injuries to which the spine is liable, dwelling particularly upon the necessity of our inquiring minutely and circumstantially into the manner in which the accident has occurred: as, whether from a weight from above crushing down the patient till the spine has given way; whether the patient has had the back bent, and the spine driven against a projecting stone; or whether he has been struck upon the part? The answers to these questions tending to show how far the symptoms may fairly be attributed to the shock given to the spinal marrow; for, if this be the cause of the palsy of the limbs, the prognosis, of course, is favourable: but, if there be fracture, the prospect becomes much more gloomy.

"If the bone is broken, there is still hope that it may be the spinous processes which are fractured and bruised; but, when it is known that the body or arch of the vertebræ is broken, then the case is almost desperate. Yet observe that, when I use this word, I do not authorise you to follow a line of practice which is to extinguish all hope. The immediate causes of death from such a condition of the spine are, first, inflammation of the membranes of the spinal marrow, and, secondly, contusion or rupture of the spinal marrow."

We now come to that portion of the Lecture which is devoted to a critical examination of the opinions on injuries of the spine, contained in Sir A. Cooper's recent work. Of these opinions we formerly gave a very detailed account, and our limits prohibit us from again dwelling upon this part of the subject: we shall merely remark, that our readers will find, on consulting Mr. Bell's work, many important and novel observations concerning practical points, and numerous examples of what he alledges to be mistakes made by Sir A. Cooper, not only with regard to his pathological reasoning, but even respecting the nature and treatment of some of the most frequent injuries of the spine. But, as we cannot do justice to either party within our restricted limits, we shall pass on to that which seems to us of most importance: we allude to the question of trephining the vertebræ, which we shall give at full length.

"Before I proceed, I am bound to remind you of a fault, which I must presume you have in common with other young men educated to surgery. You are anatomists and practised in dissection, and thence arises an intolerable itch to be doing, and a fondness for operation, and an admiration of what is called bold surgery. I say it is my duty as a teacher to restrain this disposition, not to add to it; but to substitute just and humane feelings. We are about to examine a new invention, a proposal to trepan the spine. This operation is proposed on an analogy with the trepanning of the skull."

After showing the mistakes which have arisen in consequence of confounding *compression* with *irritation* of the brain, he proceeds:

"Analogy is a very natural method of illustrating a medical subject, but it requires a delicate care that the points of resemblance be just. With respect to the treatment of fracture of the spine, says Sir A. Cooper, nothing has hitherto been effectually done in surgery; and, to fill up this hiatus, he proposes to trepan and raise up the depressed bone. I know not by what strange inconsequent reasoning he conceives that he is successfully advocating this practice, when he asserts that a fractured spine may unite again: we admit the fact; for here is a preparation before us, in which the fractured tube of the spine had been reunited. [*Mr. B. here refers to one of the plates.*] But it is beyond my comprehension how it should ever occur to him, that, because the spine may be reunited after fracture, he should therefore be at liberty to cut down through the integuments and muscles, and use the trephine, and take out the portion of bone, and expose the membrane of the spinal marrow.

"Sir Astley Cooper has been accustomed, in the discussion of this and other subjects, to make use of language in allusion to me, which, although it cannot betray me into a retort unbecoming my situation, at least sets me free from that delicacy and reserve, and those restraints, which I might otherwise have felt, and in your presence always have studied, in treating this gentleman's doctrines. It is language which is intended to find its way to your ears, and, seeing from whom it comes, might in some degree influence you, were I not patiently to refute the opinions with which it has been made to stand connected. In what he is represented to have said of the objections stated to this operation, which he so unadvisedly recommends, Sir Astley pretty obviously alludes to my Hospital Reports. Now let us lay aside all consideration of the language and tone of what he has said, and attend only to the principles on which the practice must rest.

"'If you could save one life in ten,—ay, one in a hundred, by such an operation, it is your duty to attempt it, notwithstanding any objections which some *foolish persons* may have urged against it.' He continues in the following extraordinary strain: 'Suppose any one now present were in this state himself,—suppose him put to bed with paralysis of the lower extremities, and fully acquainted with the inevitable result if nothing were done, would he not be glad to have any attempt made to save him? would it not be foolish and unmanly to say he would rather die than have such an attempt made? The operation is not severe, it cannot add to his danger; and as to the pain, no man, who is a man, would regard it. In the two cases in which the attempt was made, the operation did not shorten life; on the contrary, there is reason to believe that it prolonged it. You will be justified, therefore, in making the attempt. Though I may not live long enough to see the operation frequently performed, I have no doubt that it will be occasionally performed with success. There is no reason why it should not; and he who says that it ought not to be attempted, *is a blockhead, (a laugh).*'

"I should almost be tempted to believe that some enemy had done this for Sir Astley Cooper, were it not consistent in its tone with that also published in his quarto volume, when speaking of the same subject: 'Nothing is so easy as to condemn others: but be it remembered that the disposition to do so is a proof of a weak head and a bad heart; and that it is always to be discouraged in a profession where character is all in all.' (*Treatise on Dislocation*, p. 560.)

"We must submit to hear many strange proposals for the improvement of our profession, in the present day, from young men ambitious of notice; but that a man of Sir Astley's years and station should talk as he has done before students, and give them his authority for laying a patient upon his belly, and by incisions laying bare the bones of the spine, breaking up these bones, and exposing the spinal marrow itself, exceeds all belief. If it had been merely proposed to make such an incision of the skin, as would have allowed us with forceps to pull out a portion of the spine which had been driven in, I should only have said, 'the operation will be fruitless:' I should not have thought that it deserved severity of criticism. But then there would, in this simple practice, have been no operation; nothing bold; nothing striking from its novelty. We might have almost considered it as a natural proceeding, though, I am afraid, not a successful one. But the bone must be trephined! Now let us examine why? Why is it that you apply the trephine to the skull? Is it not because you cannot raise the bone without it? You have nothing to lay hold of with the point of your elevator. And, if you were even to raise the broken portion, you could not bring it away, because the depressed fragment is larger than the diameter of the hole. But, if the ring of the vertebræ be broken down, where is the necessity for applying the trephine? You have the projecting spinous process; you have the inferior and superior edge of the bone on which to place your elevator, or to lay hold of it with your forceps. I cannot, for the life of me, imagine any reason why that ring of bone should be trephined, and in two places.

"Gentlemen, before you bow to this authority, consider the subject under two aspects: first, as you stand by the bedside of your patient, pondering on his condition; and, in the second place, reflect whether you are required to give your assent to the statement of a just analogy.—A man has received an injury on the spine; his lower extremities are motionless; you suspect that the bone is broken. Aware, in these circumstances, of the danger of bruising or pricking the spinal marrow, you only gently incline the body, and feel the part. It is painful and tumid, and beneath the swollen integuments you think you can feel the spinous process crushed. You know well that you ought not to press and move the part as you might a broken radius. What then, let me ask, are your reflections? 1. That this may be concussion only. 2. That the spinous processes may be broken. 3. That extravasation may be in part the cause of the symptoms. 4. Anxious to inquire out the nature of the injury, if it appears that the column has been bent or twisted, you fear that it may be a fracture of the body of the vertebræ. Such are the questions you put to yourself; such the difficulties of the diagnosis. You cannot determine whether the man suffers from concussion, from extravasation, from fracture of the tube or of the body of the vertebræ; or whether the spinal marrow be compressed, or torn, or crushed wholly, or divided?

"Now the question is, should this man be turned on his belly, and a portion of the tube of the vertebræ be dug out? Do not conceal the fact from yourselves. Try the operation on the dead body, and then judge of the degree of violence necessary to its accomplishment. One gentleman tells you, 'the operation is not severe, it cannot add to the patient's danger.' By this we may know what are his notions of a severe operation. This is the report of which he approves, and which he offers as an example: 'He made an incision upon the depressed bone, as the patient was lying upon his breast, raised the muscles covering the spinal arch, applied a small trephine to the arch, and cut it through on each side, so as to remove the spinous process and the arch of bone which pressed upon the spinal marrow.' *(Treatise on Dislocations*, p. 559.) The man must be already dead whose condition is not made worse by such an operation as this! What sort of schooling must he have had, who does not believe that a man would be the worse for having the bone dug out from around the spinal marrow? If the theca were entire, it would be so far favourable; and yet we have seen the consequences of diastasis. If the sheath were perforated, it would be more quickly fatal: inflammation would presently commence, and the patient would be carried off, with symptoms such as I have described to be consequent on inflammation of the spinal membranes.

"There is an expression used which requires some comment. The same authority has it, that, in two instances in which his pupils have operated, no harm resulted, but, on the contrary, good. It is not meant by this that the patients lived, but that the symptoms of paralysis were relieved. I have seen many deceived by the same occurrence after operations on the skull; and it is very proper that you should know the reason of a seeming amendment, when there is no reasonable hope." (P. 17—22.)

Mr. Bell proceeds to show that the apparent amendment after this perilous operation depends on the excitement produced; and he illustrates this position by alluding to the deceptive appearances of improvement which not unfrequently result from trephining the skull. Indeed, we are satisfied, on reading the cases related by Sir A. Cooper and Mr. Tyrrel, that symptoms similar to those which these gentlemen regarded as proofs of the partial success of the operation, are frequently to be found in cases of fractured vertebræ, where no such operation has been performed, nor any attempt made to raise the depressed portion of bone. The language used on this subject by Mr. John Bell, in his "Principles of Surgery," (vol. i. p. 626,) is exceedingly strong, and merits the more attention when we consider that timidity was no part of his character; and that, on the contrary, the tendency of his writings is eminently qualified, and no doubt has partly contributed, to give to surgeons of the present day that boldness in operating, which seems in many cases to be carried to the extreme. His words are,—"Notwithstanding the bloody operations described in books, of making incisions, finding the fractured or luxated bone, and drawing it out by the spines or splinters, there is nothing practicable; and those very ignorant directions, given upon the highest authorities, are dangerous to none but boys. The cutting into the fractured vertebræ is a dream."

The first Lecture concludes with the following words:

"I have now placed before you a suite of preparations, such as I believe no private collection can boast of; with one or two exceptions, they occurred under my own observation: they make the subject complete,—that is to say, they afford sufficient data on which to reason safely; and it is to me very gratifying that, to taunting, I can oppose facts and substantial vouchers, which require only to be contemplated and understood in order to carry conviction. But I have no doubt that you have already made up your minds upon this subject: you perceive that, when called to a person who has received an injury of the spine, there must be much difficulty in ascertaining the degree and kind of injury. He may be paralysed with fracture; he may have suffered fracture without paralysis; he may be paralysed independently of fracture. Concussion may be the cause of symptoms, extravasation may be the cause of symptoms. With strong suspicions of fracture, you are not to turn the patient, and to press and examine, to satisfy your mind, if no plan of treatment is to be directed by the result of that examination. You are in no case authorised to cut down upon the bone, and to trepan it, and to expose the spinal marrow or its sheath. I have earnestly assigned the reasons against it, and in opposition to crude mechanical notions. Our whole attention must be directed to preserve the spine at rest, and to ward off the rising inflammation. Do this, and follow as bold practice as you choose."

The second Lecture, on Injuries of the Thigh-bone, has for one object to show the importance of combining anatomical demonstrations with surgical principles. Here again our limits restrict us to a very cursory notice of the observations made by Mr. Bell, with respect to fracture and dislocation of this important part; though our readers may form some idea of their extent when we inform them that there are no less than twenty-four figures, each in illustration of some practical question. After describing a variety of specimens, which prove that the nature of an injury differs in its characters according to the particular part of the bone which is affected, the author enters into the question of the propriety of amputating at the hip-joint,—especially in cases of necrosis. This question he answers decidedly in the negative, although he does not go so far as to say that no case can arise requiring amputation at the hip-joint. In stating this, however, we would not be understood as expressing any opinion: our readers are probably aware that this formidable operation has been successively performed by Mr. Emery, Mr. Brodie, Mr. Guthrie, Mr. Symes, and Sir A. Cooper; and, as Mr. Bell justly remarks, "a groupe of more respectable names cannot be placed together."

After some remarks on diastasis, we find the position in which the limb ought to be placed when the shaft of the bone is fractured, thus discussed:

"The size of the femur, and strength of these processes for the attachment of muscles, give sufficient proof of the difficulties which the surgeon has to encounter in fractures of this bone, and of the powerful retraction and distortion which he must find the means of counteracting. I now place before you eighteen specimens of thigh-bones fractured at the central portion. Of these, sixteen are distorted from the natural form in the same direction; that is to say, the superior portion is drawn forward and upward, and the inferior relatively depressed. [*Mr. B. here refers to one of the plates.*] This should remind you that there is, in one sense, no accident in this distortion: that the powers acting on the limb are in their operation as certain as the direction and insertion of the muscles are uniform. Had I shown you only one specimen, you would naturally have concluded that it was an accidental distortion. But you see that it is a necessary result of the form of the bone and the operation of the muscles, as a person in sleep draws up the thigh to the belly by the prevailing action of the psoas magnus and iliacus internus. In the erect posture, these muscles sustain the trunk upon the head of the thigh-bone; and, when lying supine, the weight of the thigh counteracts them, and they are in some measure stretched: but, when the thigh-bone is broken in the middle, or when it is cut through in amputation, the upper portion of the bone is immediately erected or drawn forward; and the surgeon's attention must be engaged, not to depress the bone, for that produces excitement and spasm, but to counteract its influence in another manner."

He proceeds to illustrate the manner of laying the thigh-bone on the inclined plane, by the natural position of the stump after amputation; and this leads him to comment on the observations made Sir Astley in his work on Dislocations. This gentleman, according to Mr. Bell, has had the old machine for fractures of the *tibia* engraved, in order to show that he has laid the fractured *thigh* upon an inclined plane.

"I believe," says Mr. Bell, "the double inclined plane to be a most useful and necessary contrivance, whether it is his or mine, or some one's else; but I am quite sure the principle on which I use it is doubly interesting, and that it is applicable to various circumstances of disease, accident, and operation. Here let me remind you, that when you place the thigh in the position on the double inclined plane, you ought not to forget the common means of setting fractures. I have seen some of my own pupils lay the fractured limb upon the double inclined plane without splint or bandage. Now a splint ought to go down from the hip, along the outside of the thigh, to the knee. You see here the strength of the external trochanter, and therefore can justly estimate the power of the muscles inserted into it. These muscles, especially in a high fracture of the thigh, will draw the head of the bone outwards: a splint will in some degree restrain that; but, if the line in which the thigh lies be a little inclined outwards, and the body of the patient raised, it will also serve to keep the extremities of the bone in due apposition."

Our readers may, perhaps, be surprised that they have not yet found in our analysis any notice of the question at present so much at issue, respecting fracture within the capsular ligament of the hip. We now come to this discussion, and shall quote without comment, leaving our readers to draw their own conclusions:

"We now come to the consideration of the neck of the bone; and I

shall, in the first place, give you a sketch of what has been done on this subject by my brother, Mr. John Bell. Here let me observe, that his four large volumes of the Principles of Surgery are not suited for your reading until you are well initiated in your profession, and even then they require a commentator; but, on the other hand, it will be a book valuable in succeeding ages, and to all surgeons who are desirous of seeking the principles of their profession. Not only the ingenuity is to be admired which he displays upon this very subject, but also his felicity in illustrating the principles. Among other things, he explains how it happens that the neck of the thigh-bone is abruptly broken off within the capsule, or, again, that the head of the bone is extensively fractured, and the portions thrust among the torn membranes and muscles; and he explains at great length, and with every possible variety of illustration, how it happens that the one is curable, and the other incurable. He proceeds thus, page 550: 'When a bone is broken, the soft parts are thickened around it; there is a general soft swelling of the limb, accompanied with a particular tumor surrounding the part, which tumor is hard and firm, and feels as if there were formed round the bone a gland-like mass, for the purpose of generating callus. When we break the leg of an animal, and examine this thickening, we find the muscles, the cellular substance, and the periosteum, thickened, and firmly adhering to the ends of the broken bone: the part is very vascular, and it would appear that this turgescence, swelling, and high action of the vessels, were determined to the generation of bone, which being generated, the action subsides, and the swelling and thickness dissolve.' He continues at page 551: 'But, when the rotula or knee-pan is broken, we have to do with a bone which is in very different circumstances." And at page 553: 'The neck of the thigh-bone, which is completely insulated in its natural condition, can form, when broken, none of those conditions with the surrounding parts which should help to make up a mass capable of retaining the bones in close contact, and of assisting in the generation of callus. This is the reason why all our ingenuity is exhausted in vain,—why each successive generation has condemned the inventions of the preceding age. All our hopes of succeeding in the cure of fracture in the neck of the thigh-bone have been successively abandoned, and we are almost persuaded to subscribe to the bold, unlimited affirmation of Platner, "Nunquam os eâ parte glutinari posse nec membram in antiquum statum reverti." The mechanism I have explained is, I fear, a true answer to the question of the celebrated Dessault, "Why should not the neck heal as well as any other part of this bone?" But why is the neck of the thigh-bone, when it does reunite, surrounded with so clumsy a mass of callus? This also must be explained; for it is a fact, and an interesting one, and must have a place in the account which I am presently to give of the various conditions in which the fractured thigh-bone is found after death.'

"We see by these excerpts the course of his reflections. What will you say, then, when you find that those ideas, so ingenious and so judicious, are extended and illustrated by narratives and cases and marginal sketches, forming a considerable portion of an elegant work dedicated to Sir Astley Cooper himself? That gentleman must have had great reliance upon the indolence of the part of the profession to which he addresses himself, when he could compose, I may say, two large volumes, in which the fracture of the neck of the thigh-bone forms the most prominent discussion, without a word of this matter."

We next find some remarks relative to fracture of the patella.

"In treating of the fractures of the neck of the thigh-bone, our modern authorities have copied Mr. John Bell in illustrating the subject by the condition of the fractured patella; and here, too, they have been negligent of facts, and very inaccurate in their mode of reasoning. They have too hastily concluded that the patella did not unite by bone. This very week a woman goes out of the Middlesex Hospital with the fractured patella united by bone, and you can feel the ridge of union.* Admitting that we may be deceived in this, there can be no deception in the preparation which I place in your hands: you have the patella shattered and reunited by bone, and you perceive the fragments are united with perfect regularity. What are we to make of these facts, when we also take into the estimate the opinions of Sir Astley Cooper and his commentators? Shall we allow the medical profession, extended as it now is, and forming one body, not in London or England, but in Europe and America, to suppose that there is but one school and one opinion here, and that our opinions are unsettled on a common matter of observation and of daily occurrence? I must once more explain this.

"In the common case of fracture of the patella by the sudden action of the quadriceps extensor, the bone is broken, and the pieces drawn separate, without that degree of violence which is necessary to produce reunion by bone. But when the patella is broken by a blow upon it, as by the kick of a horse, there is not only less retraction, but the injury, bloody effusion, tumefaction, and rigidity of the parts, resemble that which attends the fracture of any other bone, and the fragments unite by bone.

"I have here beside me eight specimens of fractured patella reunited by ligament, and two by bone: were I to present to you those only which are united by ligament, (and which are so common that we disregard them, and cease to collect them,) we should fall into the error of the day, and conclude that the knee-pan does not unite by bone, and that it is a provision of nature that it should not, lest the joint should become anchylosed or intercepted by the processes of new bone. But the ninth specimen decides the matter. You see that the fracture is not merely across, but that there has been a rent longitudinally; that the bone has been shattered, and therefore that it united by bone.

"As to the head and neck of the thigh-bone not having sufficiency of vessels to take upon them the ossific action, it is an idle theory. It is, indeed, amusing to see how the matter-of-fact man riots and luxuriates in a theory when he can. It has not been proved that absorption and wasting of the bone is an operation requiring less activity of vessels than the secretion of bone. They have never inquired whether it is the loose head or the extremity of the bone that wastes, or whether they be

* "Bolton came in with fractured patella 27th December, and was dismissed 10th March."

equally wasted. They have not proved that the union by bone to the socket of the os innominatum, which sometimes takes place, requires less activity of vessels than its union to the extremity of the femur; and yet it has been thought conclusive to say, that 'the third and principal reason which may be assigned for the want of union of this fracture, is the absence of ossific action in the head of the thigh-bone, when separated from its cervix.' They have not leisure to observe the facts; they pretend to despise all authority but that of a London hospital surgeon, conclude that Latin is used to cloak a lie; and they do not reason correctly on what is passing before their eyes. The notes or heads of subjects on Sir Astley's margin might, with no loss to the reader, be transferred to the pages of Mr. John Bell. The opinions are the same; but the reasoning and the illustration, as might be expected, are somewhat different. Thus, the want of union is attributed to the want of apposition. This is in part true, but this is not the question at issue. We do not need to be informed that bones far apart do not unite; but we do require to know why the bone which I place in your hands is not reunited. [*Mr. B. here refers to one of the figures.*] The cervix of the femur, you see, is absorbed, the head of the bone has come into close contact with the extremity of the shaft; and yet there is no union by bone, but only a membrane between them. It is quite clear that, if the retraction of the bone were the cause of the want of union, the distinction of cases would not be marked by so precise a line,—viz. whether the fracture is a quarter of an inch within or without the capsule of the joint.

"The real cause is one which you can best understand who see the parts before you. If the bone be broken within this capsule, it is attended with an increase of colourless effusion into the joint, and the bones remain loose and subject to motion. But, if the bone be broken external to the joint, the cellular connexions are torn, and there is bloody effusion: there follows this—inflammation and consolidation of the surrounding parts; the bones are sustained by this mass of inflamed matter; and in due time bone is formed in it, and that bone constitutes the medium of reunion.

"That I may not omit the rule of practice, allow me to state that, when you are by the bedside of the patient who has fractured the neck of the thigh-bone, you cannot decide with absolute certainty whether the bone be broken in such a manner as to unite or not. You are therefore to lay aside these questions, which are for the time so strenuously advocated, of fracture within or without the joint, and to proceed upon the supposition that the bones may admit of reunion. The limb is to be laid on the double inclined plane, and a belt and compress is to be put round the pelvis, so as at once to offer some resistance to the rising of the trochanter, and press the broken surfaces together. But, if it appear that, after six weeks, there is no reunion, nor such stiffness and swelling as forbode it, we must let the patient rise and use a crutch, lest the parts fall out of use and waste."

Our author next makes some important observations on consecutive dislocations, and on the very common symptom of one leg appearing shorter than the other in cases of injury of the hip-joint. Alluding to Sir A. Cooper, he says—

"After examining a preparation of a fracture of the neck of the thigh-bone within the joint, he sends for the shoe which the man had worn, and finds, by measurement of the heel, that the bone must have been shortened four inches, since the additional heel to the shoe of the lame leg, and the padding within, amounted to this. Reflect a moment on the process of reasoning here: measure the length of the cervix, estimate its obliquity, and you will find three-fourths of an inch in the distance betwixt the hip and the knee results from the neck of the thigh-bone; and, notwithstanding the obvious conclusion from this, we are informed that the absorption of the neck of the bone shortens the limb four inches. What a precious thing it is to have to depend on a matter-of-fact man, who will be contented with nothing but actual admeasurement! You must have seen an unfortunate cripple walking or hobbling with his stick, his hand pressed against the spine of the ilium, and the pelvis and the whole body inclined to the other side, to ease the tender hip-joint. It is this inclination which appears to shorten the diseased limb to a degree much beyond the actual diminution of its length. I verily believe that the gentleman who sent for the shoe, to know how much the limb had been shortened by the accident to the hip-joint, had never thought whether the near or the off wheel of his carriage bore the greater share of his weight; and yet the one question involves the other, —since he that does not know the principle on which the carriage-wheel is constructed, can know very little of the neck of the thigh-bone; but there are some gentlemen who will know nothing unless it be practical."

We have now extracted from these Lectures such facts as we have thought most likely to excite interest; and we repeat, that, although we lament the cause of this controversy, we cannot but hope that it will give a stimulus to the students in surgery, which must be attended with benefit; and that it will induce them to look back into the records of our art, in which many valuable doctrines and practical lessons are allowed to remain in utter oblivion, and of as little use to the profession as if they never had occurred. We take leave of the subject in the concluding words of the author.

"I hope you believe me incapable of saying any thing to you, which I should wish to conceal from the seniors of the profession, or the public in general. What I have delivered to you I mean to publish; and, if you shall find any difference in what you shall afterwards read, I trust it will only be in the greater distinctness and force of the language, since I feel restrained in delivering myself here, where those who may think themselves affected by my sentiments cannot offer their vindication. In what I have been forced to deliver, I have had no personal or hostile feeling. Expressions which might give me a momentary feeling of irritation, or might naturally have excited a hasty word in return, could never influence me long enough to lead me into the detail into which I have gone to-day.

"As to Sir Astley Cooper, I know that I shall not hurt him; neither diminish his opportunities of doing good, nor mortify his feelings. He is armed in double proof. Indeed, he has said, in the very book which I have referred to, on the occasion of a gentleman criticising him, 'that it has led him to think better of his work than he had previously been disposed to do.' This is some relief to me in concluding the observations which I have been called upon to make; and I most sincerely declare, that I shall be happy to correct the general impression on these matters, without giving Sir Astley Cooper one moment's uneasiness, or withdrawing him for the shortest time from his pursuits. But I wish to state decidedly to you, and to the profession generally,—nay, I wish to do more, I wish to put it upon record,— that all his contemporaries were not carried along with him; that those who shall succeed us in our hospitals and our lecture-rooms may know that I entertained opinions very different from his on many important practical questions. I have gone thoroughly into one subject, that I might be relieved from the necessity of speaking of others. The profession, or that small portion of it who value my opinion, shall know by this my idea of his capacity for settling the rules on delicate questions of practice; but I shall not be easily drawn to say more than duty requires. I have met him on his own ground; I have not gone into vain criticism of language, nor have I found fault needlessly; I have opposed him only as a practical hospital surgeon."

---

ART. II.—*Transactions of the Medico-Chirurgical Society of Edinburgh; instituted August* 2, 1821. With Plates.—8vo. pp. 697. Edinburgh, 1824.

[Concluded from page 417.]

*Cases of Infantile Disease, in which Erosions and Perforations of the Alimentary Canal were found after Death; with Remarks.* By JOHN GAIRDNER, M.D. &c.

IN our Number for November 1822, we drew the attention of our readers to the work of M. CRUVEILHIER, on Softening of the Stomach and other parts of the Alimentary Canal in Children.* The description of a similar disease forms the subject of Dr. GAIRDNER's paper before us. The author is of opinion, 1st, that erosions and perforations of various parts of the alimentary canal, particularly the stomach, frequently occur in children who do not appear to have suffered from any disease of those parts,—in other words, that these erosions and perforations are not the result of ulceration, but are effected by the solvent powers of the juices; and, 2dly, that similar appearances are found in the bodies of children whose symptoms are such as to mark unequivocally some disease of the stomach and bowels, by which such an organic change is produced as renders the parts

---

* *Medecine pratique eclairée par l'Anatomie et la Physiologie Pathologique.* Par J. CRUVEILHIER.

more easily acted upon; but that, in this case likewise, the erosions, &c. take place after death. Of these positions, the two following cases may be taken as illustrations.

"Case I.—On the 8th of August, 1821, I was invited by Mr. Bathgate, of St. James's-street, to assist at the examination of the body of a female infant which had been under his care. I had not seen it during life, but I learned from Mr. B. the following particulars:—It was thirteen months old, and had been troubled with cough from the preceding January. About three weeks before it died, it became very feverish, with a pulse at 160, and breathing about seventy in the minute, but without much heat of the skin, which was frequently colder than natural. It expectorated a great deal of matter with its cough, and had frequent sweatings, which alternated with diarrhœa. This last symptom began three weeks before its death, and about the same period it became subject to occasional vomiting. Among the matter vomited, there was generally a quantity of the purulent expectoration which had been previously swallowed. The child, during these three weeks, did not lie on the left side, but invariably turned to the other, and a degree of swelling was perceived in the left hypochondrium. For the last ten days it was unable to suck, apparently from debility, but had an insatiable thirst, and drank water and other liquids with eagerness. It slept a great deal, but was easily disturbed, and very fretful, often crying without apparent cause. The matter evacuated from the bowels was of a greenish colour.

"*Dissection.*—The lungs were much diseased, and full of tubercles on both sides and in every part of their substance. Some of these on the left side had suppurated extensively, and burst into the bronchial tubes, which on that side were completely full of pus. A portion of the left lung sunk in water, and the whole of it must have been for some time totally incapable of performing its ordinary functions. The contents of the stomach were effused into the abdominal cavity, through a large opening in its left side, at the part where it is in the natural state connected with the spleen. The edges of the opening were ragged, soft, and extremely thin. There was a slightly reddish tinge all round the perforation, forming as it were a narrow border, not so much as a tenth of an inch in breadth. With this exception, there was no appearance either in the stomach or neighbouring parts, indicative of vascular turgescence, or inflammatory action. The opening was so large as to admit four fingers of my right hand.

"It is evident that in this case the pulmonary complaint was the primary disease, and that this singular state of the stomach was only accidentally conjoined with it. The child's father died phthisical only six weeks before it. It was nursed by its mother, whose health was good during the time of nursing. In the early months of its life, the child was stout and plump." (P. 312—314.)

"Case II.—This child, a boy, was nursed by his mother, who enjoyed good health, and had a plentiful supply of milk. The child, though by no means robust, had every appearance of thriving till it was about eight months old. At this time it was seized, one evening, with a fit of violent crying. This occurrence in a child, which was at other times habitually placid and contented, naturally arrested attention; and, as the whole family had made arrangements to depart the following day for a distant part of the country, I was consulted with regard to the propriety of undertaking the journey. The child soon after ceased crying, fell into a profound sleep, and awakened the following morning quite well. As they proceeded to the country, I did not see the child for some time; but its mother informed me, that from that period it seemed to her to have a less healthy look; that it frequently had an expression of dissatisfaction depicted in its countenance; and that it was keenly sensible to the slightest impression of cold. I shall not now discuss the question, whether these changes are to be considered as indicating the first approaches of the disease which afterwards proved fatal, or whether they arose simply from the irritation of dentition; though I may state that I incline to the latter opinion.

"On the 31st of May, 1822, having completed his eleventh month, and got two of his incisors, he was deprived of the breast. On the same day, before the change of food could have had time to produce any impression, he was troubled with a trifling degree of diarrhœa. This complaint went on, but was so slight for a week as not to make any one uneasy about him, and was treated by regulation of the diet, without medicines.

"On the evening of the 8th of June, he had half a grain of calomel, with three grains of the prepared carbonate of lime. This was repeated on the 9th, and operated with considerable power on the bowels. I divided the gums over some of the incisors, which were near to cutting; and on the 10th, the bowel complaint still continuing, he was ordered a powder consisting of eight grains of prepared carbonate of lime, two of rhubarb, and two of Dover's powder.

"On the 11th, in consequence of the occurrence of a fit of vomiting, and the continuance of the diarrhœa, he had one-fourth of a grain of opium made into a draught.

"On the 12th, stomach still irritable, but less so than the preceding day; diarrhœa checked in the night, but returned in the morning; no appetite; much thirst. Emaciation and loss of strength now very evident.

"A degree of sinking of the eyeball within the orbit had been remarked for a considerable time, both by me and by the mother of the child, who, upon my mentioning it, observed that she thought it natural to him; but it was now evidently increased. In other respects the eyes presented no unusual appearance; the pupils were perfectly contractile, and the child quite alive to all external impressions. Pulse natural, as it had been from the first. No heat, either general or local, nor any other febrile symptom. A mixture, consisting of rhubarb and chalk, with a proportion of opium, was directed to be given, in a proper quantity, three times a-day.

"14th.—Child languid, and exhausted to a very alarming degree; ardent thirst, some retchings; frequent unrefreshing slumbers, from which the slightest touch awaked him.

"15th.—A number of aphthous specks were observed on the tongue. Stomach less irritable, but incapable of receiving food. The other appearances as formerly.

"The specks consisted of a soft matter, which was easily rubbed off. No ulcerations appeared on the surface of the tongue after their removal.

"The diarrhœa and emaciation continued progressively to increase, till the child sunk from exhaustion. Its death happened on the evening of the 17th, seventeen days from the commencement of diarrhœa, and seven from the first occurrence of vomiting. Nothing happened during the last two days worthy of particular mention, except the appearance of the stools, which consisted of mucus in large quantity, mixed with a proportion of bile, and on one or two occasions streaked with blood. As the diarrhœa was particularly severe during the two last days, powerful opiates and astringent enemata were unavailingly resorted to.

"*Dissection.*—On opening the abdomen, the viscera appeared at first sight to be all sound; but, on slitting up that part of the omentum which joins the great curvature of the stomach to the transverse arch of the colon, the contents of the stomach were seen pouring out through several perforations in that viscus. These perforations were four in number; they were situated on the posterior surface of the stomach, not far from its splenic extremity; they were nearly of the same size, the largest scarcely big enough to admit the point of my fore-finger. They were separated from each other by portions of the substance of the stomach, not so broad as the apertures themselves, and these portions were in an exceedingly soft state, and very thin. More of the internal coats appeared to be destroyed than of the peritoneal. No vascular turgescence, no adhesions, nor other marks of inflammation, were discoverable. The gall-bladder was full of bile. The intestines appeared thin and semi-transparent, partially distended with air, and strongly tinged, in one or two places, by transudation of bile: they contained scarcely any solid or fluid matter. The liver, mesenteric glands, and all the other viscera of the abdomen, were natural; so also were those of the thorax. In short, the disorganization of the stomach constituted the only deviation from the healthy structure." (P. 316—320.)

On turning to our analysis of Cruveilhier's work, our readers will find that he insists more especially upon the conversion of the parts into a soft gelatinous mass: indeed, he asserts that what he calls acute perforations *(aiguës,)* are always preceded by this peculiar change and by thickening of the walls of the organ. It is remarkable that, in all the cases of perforation described by the French pathologist, no change of colour nor injection of vessels was observed in the contiguous parts. Dr. Jaeger has related a considerable number of similar cases of softening, in Hufeland's Journal for 1811 and 1813. These, with Cruveilhier's, his own, and some others, are ranged by the author in two tables; the former containing twenty-one, and the latter twenty-three cases.

The arguments which have induced Dr. Gairdner to adopt the opinion that these perforations, when they occur, are not the effect of any action carried on during life, are as follow:

"1st, The human stomach has been found perforated in persons who have died a violent death without previous illness; and in whom, therefore, it is to be presumed that no such important lesion of a vital organ had existed prior to death. I need scarcely add, that this statement rests on the authority of Mr. John Hunter, who mentions three cases of perforation of the splenic extremity of the stomach, in two of which death was produced by injury of the head, and in the other by strangulation.

"2d. Similar observations, corroborating this inference, have been made on the stomachs of the inferior animals: on those of fishes, by Hunter, Spallanzani, and Dr. Joseph Adams; on those of rabbits, by the latter, and also by Sir Astley Cooper and Mr. Carlisle; and on that of a dog, by Dr. Adams. In all these cases the animals were previously healthy, as far as could be ascertained, and were put to death by violence. I have myself witnessed similar appearances.

"3d. In cases in which a perforation of the alimentary canal has occurred, erosions of the surfaces of the adjoining viscera, softening and partial solution of their substance, erosions of the abdominal parietes, and erosions and perforations of the diaphragm, with effusion of the contents of the stomach through such perforations into the cavity of the thorax, have all been occasionally met with. No symptoms have been observed in such cases, from which any injury of these important parts could have been surmised. Appearances of this description are mentioned in Mr. Hunter's work above cited; two instances have already been quoted from Jaeger; and similar facts are recorded by Professor Chaussier, Mr. Allan Burns, and more recently by Professor Haviland, of Cambridge.

"4th. In the stomach of a fish, which was perforated at one extremity, Dr. Adams found a smaller fish partially digested, and a live worm quite entire. The worm was killed, and, after having been a few hours re-immersed in the fluids of the stomach, was found 'more than half digested.'

"5th. The theory derives further support from the cases already adverted to, of perforations of the stomach and bowels in the bodies of persons who have died of other diseases; since it is not easy to explain why any disease of the stomach or bowels should effect such extensive destruction, simultaneously with pneumonia, apoplexy, or convulsions, and that without exhibiting any symptoms calculated to excite suspicion of its presence.

"6th. Mr. Allan Burns, of Glasgow, after exhibiting to his pupils a perforation of the stomach, and pointing out to them that the liver was sound, replaced the parts in situ, and sewed up the body. On opening it after two days, he found that the peritoneal coat of the liver opposite to the opening in the stomach, was completely dissolved, and that the liver itself was tender to a considerable depth." (P. 348—351.)

The symptoms observed during the progress of the disease were—diarrhœa, with green watery stools, accompanied with vomiting and great thirst, but without heat of skin or increased arterial action; somnolency, from which, however, the child was roused by the slightest touch; mental acuteness; and, immediately before death, coldness of the extremities, profuse perspirations, and a fluttering pulse. Unfortunately, little that is satisfactory can be said with regard to the treatment, though both M. Cruveilheir and Dr. Gairdner think the disease is sometimes cured. The latter advises the abstraction of blood in the early stage, if the child be " at all stout," arguing that, although there be no *appearance* of increased vascularity, yet that such must necessarily take place to a greater or less extent. This, followed by a blister to the epigastrium, opiate enemata, and the warm bath, makes up the list of remedies. Among the things to be avoided are the introduction into the stomach of any food or medicines likely to produce irritation, either by the quantity or quality; such, in particular, are purgatives of every kind.

***Cases of diffuse Inflammation of the Cellular Texture, with the Appearances on Dissection, and Observations.*** By ANDREW DUNCAN, jun. M.D. &c. &c.

This paper relates principally to those interesting and hitherto inexplicable cases of inflammation and constitutional disturbance, which occasionally follow venesection, a prick received in dissection, or some similar wound. Various suppositions have been had recourse to, with a view of explaining the pathology of these: they have been successively attributed to pricking a nerve, or wounding a fascia,—to irritation of the lymphatics, or inflammation of veins. To this list Dr. DUNCAN now adds diffuse inflammation of the cellular texture. The paper occupies no less than 180 pages, and contains a detail of thirty-four cases, so that it is obvious we can do little more than point it out to the notice of those interested in the subject.

The progress of the disease varies considerably with the causes from which it has originated; but, in general, fever of the typhoid type precedes the occurrence of a diffused swelling of some part of the body, attended with severe pain and some degree of redness. Where the disease has been excited by a local injury, it generally commences at this point, and gradually extends; where it has been induced by venesection, the wound sometimes heals as readily as usual, but, for the most part, it afterwards opens again, or suppurates; more frequently it does not unite at all, but the lips remain inflamed and swollen. In the worst form of the disease, the symptoms resemble those produced by the inoculation of some virus,—a vesicle forming on the site of the puncture, with high constitutional disturbance, the inflammation spreading rapidly, and chiefly affecting the axilla, shoulder, and breast. The manner in which this propagation takes place does not seem to be clearly ascertained,—some attributing it to inflammation of the lymphatics, and others of the veins. Dr. Duncan, however, thinks both these suppositions incorrect. There is rarely any obvious or direct connexion between the injury and the inflammation of the axilla; and, indeed, so slight an abrasion is capable of admitting the poison, that in some cases no injury of any kind can be detected. In the case of Professor Dease, given in our Analysis of the third volume of the "Dublin Hospital Reports," that gentleman was not sensible of having received any scratch or puncture; and, in general, the primary wound gives little uneasiness, and excites little notice.

Such injuries, from obvious causes, are more frequent in the hands and arms than elsewhere, and the axilla is the part principally affected with secondary inflammation. This extends upwards along the neck, and downwards even to the thigh, (see Professor Dease's case,) generally confining itself to the same side; occasionally changing its seat by metastasis. The inflammation is but little elevated above the surrounding parts, shows no disposition to point, and is scarcely defined by any distinct margin; it has no central hardness, "no focus where it is most active." The general sensation communicated to the hand, on examining the part, is caused by fluid effused into the cellular texture; the swelling feels tense, and when pressed does not pit, but seems intermediate between the hardness of phlegmon, the yielding of œdema, and the elasticity of emphysema: this sensation is called *boggy* by Mr. Lizars, and *doughy* by Dr. Colles. In the case of Dr. Pett, Mr. Travers found the swelling crepitate.*

In all the cases related by Dr. Duncan, the pain was very severe: indeed, pain in the axilla is frequently the first symptom that gives alarm. The fever soon arrived at its height, but was rarely attended with coma or continued delirium. The respiration was generally more or less affected, and this depended on two causes,—viz. the painful condition of the muscles of the chest, and the extension of the inflammation to the pleura, which took place in several instances. The most favourable termination of these evils is, of course, in resolution, but this unfortunately is extremely rare; and Dr. Duncan did not meet with it in one case from local injury, after any alarming symptom had come on. The next most favourable termination is in abscess; but, in severe cases, the most common result is an extensive and diffuse suppuration of the cellular membrane. The appearances found on dissection are thus described:

"Although, in the preceding part of this essay, I have been enabled, by the kindness of various friends, to insert in detail the reports of the only series of dissections of the bodies of individuals who have fallen

---

* See *London Med. and Phys. Journal*, February 1823.

victims to this affection, or its sequelæ; yet I trust it will not be thought a work of supererogation to attempt to describe them methodically, for the true pathology of the disease can only be derived from a correct knowledge of these appearances. As this disease is progressive, affecting one part first, and then others in succession, we find it after death existing in different parts, in all its stages. Accordingly, in the part most recently affected, which was often the space between the twelfth rib and the os ilium, we find the cellular substance merely œdematous, with increased vascularity; the serum is still fluid and limpid, or tinged with red, and readily flows from the divided tissue. In a more advanced stage, the effused matter is less fluid, often higher coloured, and has not yet acquired the opacity and whiteness of purulent matter. We next find the cellular membrane gorged with a white semi-fluid matter, which does not flow from the incision, but greatly augments its thickness, and separates the particles of fat to an unusual distance from each other. In the subsequent stage, it continues opaque, whitish, or reddish, or greenish, but becomes more fluid, so that now purulent matter flows from the incision. But the pus is still contained in the cells of the tissue; and it is only in the last stage of the disease, and after the tissue is entirely broken down, that we meet with collections of purulent fluid, with sloughy membrane: even then, however, the pus is not contained in a cyst or circumscribed cavity, but is gradually lost in cellular substance in the preceding stage of the change, without any line of demarcation.

"When fluid pus is formed, I have already spoken of it as if it were broken down cellular substance; but it is perhaps rather a secretion, which is in such abundance as to rupture the cells, and break down the cellular membrane, so that the portions thus disjoined from their necessary attachments become dead, or, in common language, sphacelous. In this way we may account for the masses, like skeins of thread, drawn out of Mr. Blyth's side; and the sloughs of cellular membrane, described by Sir E. Home as resembling wet tow, and by Mr. James as looking like large wads of wet shamoy leather.

"Next to the cellular tissue, the *muscular substance* is that which is most constantly affected, although it might be doubted whether the interfibrous cellular texture alone was diseased, or whether the true muscular fibre itself was likewise inflamed. I am disposed to adopt the latter opinion, not only from the very intimate manner in which they are mixed, so that one cannot be conceived to be affected without implicating the other, but also because, on dissection, we have always found the muscular substance much more tender and easily torn than is natural, and its colour altered. In the case of Mrs. Craig, in particular, we had the most direct evidence of the ultimate destruction of both layers of the intercostal muscles; and Sir E. Home, in a rat bit by a snake, found the muscles detached from the ribs and a small portion of the scapula. In some cases the colour of the muscular fibre in the affected parts was much paler than usual, as in the body of No. 25, the last I have seen; and in others it was very much darker, as I recollect particularly in the first, that of Snell.

"The cellular membrane is abundantly supplied with *vessels* of every description, and those which belong to the diseased part cannot remain perfectly sound. Indeed, the very formation of serous and purulent fluid, which is an essential character of the disease, is the result of a morbid action of the capillary vessels; and, accordingly, we find the usual indications of what is called increased action of the vessels. The number of visible red arteries is augmented, and the veins are enlarged and turgid with black blood. These appearances of the vascular system I consider as primary. Mr. John Hunter has described a secondary affection of the veins, which has hitherto escaped my notice. 'I have found,' says he, 'in all violent inflammation of the cellular membrane, whether spontaneous or in consequence of an accident, as in compound fractures, or of surgical operations, as in the removal of an extremity, that the coats of the larger veins passing through the inflamed part became also considerably inflamed, and that their inner surfaces take on the adhesive, suppurative, and ulcerative inflammations; for, in such inflammations, I have found in many places of the veins adhesion, in others matter, and in others ulceration.' And he adds, that 'it is so common a case, that I have hardly ever seen an instance of suppuration in any part furnished with large veins, where those appearances are not evident after death.'

"The *lymphatic vessels* must also partake of the general disease, but their state has never been ascertained by dissection. The axillary glands have, however, been often observed enlarged, and imbedded in highly diseased cellular substance; but, although a swelled and tender axillary gland has been very frequently mentioned as one of the first symptoms observed, I have never found them so much diseased as at all to support the idea that their affection was the primary cause of the state of the surrounding parts. In Mr. Blyth, swellings of the lymphatic glands of the groin were amongst the last symptoms observed, and did not appear until his convalescence was advanced. In Sutherland, No. 22, a small portion of pus was found in one of the axillary glands.

"I must confess that no particular notice was, in any of our dissections, paid to the state of the *faciæ,* but at least there was no such change in it anywhere as to attract our attention. An argument in favour of the insusceptibility of inflammation as a property of tendon, may be derived from Mrs. Craig's case, in which the tendinous *septa* between the ribs were left in places where the muscular substance and all other textures had disappeared.

"The *skin* is frequently severely affected, but not essentially or primarily. This point of pathology is, I think, indisputably established by the preceding dissections." (P. 608—613.)

The *diagnosis, prognosis, prophylaxis, local and general treatment,* are severally considered; but on these heads the subject scarcely admits of any thing very satisfactory. Free incisions are strongly recommended as soon as any fluid seems to be effused.*

* Some other interesting facts contained in this volume we shall introduce in our "Historical Retrospect" next month.

### DIVISION II.

### FOREIGN.

Art. III.—*Recueil de Memoires, Consultations, et Rapports, sur divers Objets de Medicine Legale.* Par M. F. Chaussier, &c. &c.

*Collection of Memoirs, Consultations, and Reports, upon different Subjects of Legal Medicine.* By M. F. Chaussier, Knight of St. Michael, and of the Legion of Honour; Professor at the Faculty of Medicine, &c. &c.—1 vol. 8vo. pp. 519; with six Plates. Paris: Barrois and Compere, jeune. 1824.

There is no department of medical science that has been cultivated with more success, of late years, than legal medicine; and the works of Foderé, Capuron, Orfila, and Chaussier, will remain as lasting monuments of the ability and zeal with which this important subject has been treated on the Continent; whilst in our own country the productions of Dr. Gordon Smith, Dr. Paris, and Mr. Fonblanque, have lately rescued our medical literature from the charge of poverty, neglect, and barrenness, which was but too apparent before the publication of these excellent works: indeed, the whole science may be almost said to be a new creation,—at least in this country. The volume before us we consider as highly deserving of an attentive perusal, both by the public and the profession; and, if we do little more upon this occasion than introduce it to the notice of our readers, and present to them some interesting extracts in confirmation of this opinion, it is only because we intend, in the following number of our Journal, to give a more extended view of the subject, in an analysis of Dr. Smith's work.

M. Chaussier informs us that, although this volume is only published now, great part of it has been printed since the year 1816; and he makes this avowal, lest there should appear too close a similarity between some passages in his book and many that are to be met with in different theses and works already printed. "Those," he says, "who have attended to my lectures at the Faculty of Medicine in the College of France, will readily know to whom they belonged primarily."

We have no hesitation in saying that, in point of elegance of language and accuracy of expression, both with respect to the importance of the questions discussed, and the highly valuable remarks which are subjoined, that this volume holds a very high rank, and is, upon the whole, one of the most important foreign works which has come within our notice for a long time. It consists of three parts: the first treats of the mode of opening the dead body, especially in cases of judicial inquiry. In the second, numerous remarkable reports of cases of assassination, poisoning, &c. are detailed, selected from various French authors, from the time of AMBROSE PARÉ to the present day; to which are subjoined remarks upon the omissions, errors, and obscurities contained in those reports. In the third part, M. Chaussier discusses the subjects of ecchymosis, blows, contusions, bruises, &c. as applicable to medical jurisprudence. A short preface contains the history of the rise and progress of legal medicine in France; where, our readers are probably aware, it is taught by regularly appointed professors, at Paris, Montpellier, and Strasburgh.

The volume opens with a beautiful and vivid description of the dead body, the object of fear and horror to the vulgar, but so interesting in every respect to the philosopher and physician, and immediately proceeds to detail the usual manner of conducting its examination; the necessity for which cannot be better exemplified than by the anecdote related by our author, page 11.

"Two men, who had on different occasions given proofs of bearing enmity towards each other, met in the day-time in a public and well-frequented place: one of them, who was getting off his horse, and had a horse-whip in his hand, passing near his adversary, said some reproachful words to him, and, passing on, gave him a blow with his whip across the shoulders; the other, highly indignant, and furious at this unexpected treatment, ran after the one who struck him; but had scarcely gone twelve paces when he fell, and died upon the spot, pronouncing some inarticulate words. The man's death was attributed to the blow, although there was no external mark visible; and the opening of the thorax showed a great quantity of effused blood, arising from the rupture of an aneurism, a disease with which he had been afflicted many years."

The second part of this volume, namely, that more especially devoted to the detail and critical examination of various reports made upon subjects connected with legal medicine, commences with a detail taken from Ambrose Paré, and gives occasion, on the part of our author, to a digression upon the merits of that great man. We participate sincerely in the sentiments expressed by our author on this subject. For sound sense, independence of character, honesty, and candour, Paré may have been equalled, but he has never been excelled; his works deserve to be studied by all surgeons, even of the present age; they contain many inventions and observations supposed to be modern; and no one will rise from their perusal without being a better man, as well as a better surgeon. After paying this tribute to the memory of one of the brightest ornaments of the art of surgery, we pass on to the second case reported by our author, with a sketch of which we shall present our readers, in order that they may more fully appreciate M. Chaussier's method of commenting upon these reports.

"In September, 1808," (we translate M. Chaussier's words,) "a person, who was returning from a fair, where he had sold some sheep, stopped at the alehouse of a village, and fell into dispute with a man who was sitting at table with several friends. After several provocations from each party, the man who was sitting at the table rose up abruptly, and, with a knife which he had in his hand, neither very pointed nor very sharp, he aimed several blows at his adversary, which wounded him in several places,—in the back of the left hand, and at the upper part of the thorax, a little beneath the clavicle of the right side. The combatants were separated, and, when peace was restored, the wounds were examined: they bled very little, appeared but slight, confined to the skin, and even penetrating merely the surface of that. They were washed with warm wine, and, an hour after the quarrel, the wounded man went away quietly to his home, distant about half a league: nevertheless, on the morning of the following day, this man was found dead in the road. He was cold, stretched upon his face, bathed in his blood, and the limbs quite stiff. An inquiry was instituted into the causes of this man's death; the above circumstances were all stated; and the medical man, who opened the body, presented a report, of which the following is the substance:—There were two superficial wounds on the back of the hand, merely the thickness of the skin, which were covered with a layer of clotted blood, half dried; a third wound on the anterior part of the thorax, two inches beneath the sternal extremity of the clavicle of the right side, five lines in length, skin deep, and surrounded with a reddish areola, or circle, of the breadth of two lines. A fourth wound in a line with the left nipple, but nearer to the sternum in a transverse direction, two inches long, penetrating the thorax, the edges of which were gaping, without swelling or ecchymosis, and giving issue to a great quantity of blood when the position of the body was changed, or when the abdomen and thorax were pressed upon. The examination of the body showed the brain and abdominal viscera to be quite sound; but, in the thorax, several pounds of blackish blood were found in the cavity of the left side, almost entirely fluid; which being removed, it was found that the fourth wound above mentioned penetrated the thorax deeply, between the fifth and sixth ribs, directed obliquely from left to right, and from below upwards, which in its passage had perforated the left lung, divided the trunk of the pulmonary artery, a little below the curvature of the aorta, and which terminated in the substance of the right lung. The bronchiæ, trachea, and mouth, were filled with blackish and half-fluid blood, mixed with some frothy mucus. From these circumstances, the examiner drew the following conclusions:—1st. That the fourth wound was made by a *sharp* cutting instrument, flat and broad; that it was necessarily mortal; and, 2d, that the wounds of the hand were made by an instrument, not very sharp, or used with little force; that they could have had no sinister consequence, and that they had been inflicted *some time before* the penetrating wound of the thorax, since they were dried, and covered with a bloody exudation. The same remarks applied to the small wound on the anterior part of the thorax, since it was not bleeding, and surrounded by a reddish, ecchymosed areola. The justice of these conclusions was

**afterwards proved; it being ascertained that this man was met by two robbers on the road to his home, who had taken all his property, and had plunged a sabre in his chest."**

This case shows the necessity of marking, in a particular manner, the nature and character of the different wounds found on the same individual.

Several curious documents next present themselves, extracted from the works of Paré and Guillemeau; but they do not present any remarkable points of interest to the physician. We pass on to the examination of a case of supposed poisoning, taken from a work of NIC. BLEGNY, printed in 1684, and with which he was so well satisfied as to present it as a model to be followed in similar cases. The report says, that they (the examiners) were called upon to inspect the body of one Susan Pernet;—having found *all the external parts* in their natural condition, they proceeded to open the body; and, having begun with the abdomen, and afterwards opened the stomach, they found it cauterized at its lower part, *(tout cauterizé,)* which contained about an egg-full of a black gritty liquor, *(sabloneuse,)* which, being put into a tin vessel, acted upon it as acids and corrosive substances do; a small quantity of this being given to a dog, gave it great pain, as was evident *by its cries and howling :* from which they judged that Pernet had been poisoned by arsenic, corrosive sublimate, or some other corrosive mineral; and which they thought the healthy condition of the other internal parts, both in the abdomen, chest, and head, tended strongly to confirm. M. Chaussier remarks upon this case:—1st. None of the circumstances preceding death are noticed, nor the state in which the body was found; neither the age or apparent constitution, nor period of death of the party, are mentioned. 2d. Neither the form, nor size, nor nature, of that alteration which is denominated a *cauterized state* of the stomach, are described; nothing is said of the condition of the mouth and pharynx; and the state of the stomach renders it very doubtful whether every other part of the abdominal contents could be in a natural condition. 3d and 4th. The quantity of fluid found is loosely expressed; and it is absurd to judge of its nature by colour, consistence, or even its effect upon tin. 5. Neither is the circumstance of its having caused pain to the animal to which it was given, an absolute proof of the substance being a poison, and still less so if violence was used to force the animal to swallow it; for it is possible that the secretions of the stomach, altered by disease alone, may cause the death of an animal who swallows a portion of them, as has been observed by MORGAGNI : and neither the cries nor even the death of an animal are undoubted proofs of the action of poison, unless, upon opening its body, the same alterations are observable as have occurred in the person who had been poisoned.

These observations lead our author to discuss, at some length, the merits and utility of experiments made upon animals, not only with reference to the subjects before us, but also with respect to those which are made to determine some points of anatomy and physiology. M. Chaussier displays in this discussion much sound sense and goodness of disposition. We agree with him almost entirely; and we more than doubt the advantages that have been, or are to be, derived from torturing so many helpless animals, for the sake of settling some doubtful point of physiology, the application of which to practice is not only very uncertain, but in most instances absolutely unattainable. We hope that we do not go too far in saying, that, if all the brilliant experiments which impose so much upon our imaginations, and which have afforded scope for so much ingenious reasoning, had never been performed, the art of curing diseases would have suffered little or nothing. It is very rational to satisfy one's curiosity; but if that is to be done with the sacrifice of life, and the prolonged suffering of any living being, we conceive it to be unjustifiable, unless some real good is likely to be the result.

Our next extract is taken from a very long report, relating to the inspection and opening of the body of a man, who died from the wound given by a knife. It presents several points of interest, and appears to have attracted M. Chaussier's attention particularly, in consequence of its having been held out by Mahon, in his work on Legal Medicine, as a model for reports of this nature. It is, however, so long, that we can only notice some of the prominent points which our author has made the subject of his criticisms. His first observation applies to the omission of the general appearance of the body, and the time that elapsed between the infliction of the wound and death. The second which we think it necessary to notice, applies to the description of the length of the wound; in which place the reporters say that it corresponded with the breadth of the knife which the *murderer* had used: thus taking upon themselves to judge, not of the appearance of the body, but at once fixing the crime upon the accused. His next criticism applies to the great length to which the report is extended, and the needless discussions that are entered into; and he justly observes as follows:

"What was the object of the visit? To examine the condition and circumstances of a wound in the left hypochondrium, which, penetrating the abdomen, had pierced the spleen, the kidney, part of the diaphragm, and had caused an effusion into the abdomen and thorax, &c. Assuredly all this could have been said in a few lines, and in a clear manner." (P. 242.)

Another remark, which M. Chaussier makes relating to the appearances indicative of previous inflammation, is too important to omit. The redness of a surface in the dead body, he observes, is not enough to prove that there has been inflammation during life.

"The body," he says, "experiences great changes after death, and these changes, more or less rapid, depend not only on the temperature of the atmosphere, the season, and the climate, but likewise on the causes that have preceded, and the circumstances that accompanied, death. Thus, in many acute diseases, in consequence of certain violences or of the passions of the mind, the blood has lost its natural crasis; it does not coagulate, but takes a consistence resembling a brownish and soft jelly. In these cases, which are not uncommon, the interior of the right side of the heart, and the great veins, are of a red colour, more or less deep, sometimes blackish, and which is difficult to remove by washing, or even by maceration. This tint is more or less marked in all the veins which contain blood, and more especially in the stomach, the intestines, and mesentery; and in these cases also the same thing is observed in the arteries, when containing blood, but the colour is brighter than in the veins. Will it be said that these appearances denote a preceding state of inflammation? This discolouration is principally remarked at the termination of diseases of debility, in certain cases of asphyxia, apoplexy, or external violence,—in the fœtus, in the infant, as well as in the adult; and it is always more observable when the body is opened three or four days after death." (P. 245.)

We pass by the remarks upon the *proces verbal* relating the death of Mirabeau and General Hoche; though, from the latter especially, we would willingly, had our limits permitted, made some extracts. But we pause at the next case: it is a subject historically of great interest, and has occasioned much discussion. It is the report upon the death of the celebrated General Pichegru, and we shall abridge it so as to comprise, in as small a compass as possible, all the facts of the case.

"General Pichegru had been for some time a prisoner in the Tower of the Temple. On the 5th April, 1804, he was in good health, and his supper was served as usual. About ten o'clock at night his room-door was closed, the key of which was taken away. The man on guard affirms that he heard him cough and spit several times about half-past three o'clock in the morning; and at seven o'clock on the 6th, on going to light his fire, he was found dead upon his bed. A commissary of police was called, who, after a mere inspection, pronounced that he had committed suicide. A few hours after, the special criminal tribunal, to whom it had been announced that the said Ch. Pichegru *had committed suicide the night before*, appointed a commission, composed of five of its members, to make an inquiry into the fact, and to collect the particulars; and five surgeons and one physician were named to examine the body, who accordingly reported (twelve hours after the death,) that they found a body on the bed, of the male sex, apparently from forty to forty-five years of age; height, one metre sixty-eight centimetres." (Then follows a description of his features.) "After examining the general appearances of the said body, they remarked a circular impression round the neck, two fingers' breadth in extent, and more marked on the left side. That strangulation had been produced by a black cravat, tied very forcibly, in which they had passed *(on avait passé,)* a stick, forty-five centimetres in length and five round; and that this stick had been used as a tourniquet, with which the said cravat had been tightened more and more, until strangulation had been effected. They then remarked that one of the ends of this stick rested upon the left cheek, and that, in turning it with an irregular motion, it had produced upon that cheek a transverse scratch, of about six centimetres in extent. That the face was ecchymosed, the jaws closed, and the tongue caught between the teeth. That the ecchymosis extended over the whole body; the extremities were cold, and the muscles and the fingers of the hand strongly contracted; and that they judged, from the position in which the body had been found, and from the above observations, that the individual whose body had been examined, *had strangled himself.*

"The next day, the 7th of April, at nine o'clock A.M., the tribunal, by a fresh order, charged the same physician and surgeons to proceed to open the body, in the presence of certain authorities; and the following is the substance of their inspection:—In the head, the vessels, both external to the skull as well as those of the dura mater, highly injected; the longitudinal sinus gorged with blood, especially at the posterior and lower part; in the falciform process there was an ossification; the inferior surface of the brain was loaded with blood; there was nothing particular in the ventricles, excepting that the choroid plexus was of a deeper red colour than ordinary; there was an hydatid at the superior part of the annular protuberance. In the abdomen, the stomach appeared inflamed *(phlogosé)*. Both lobes of the lungs were gorged with blood, and the œsophagus was sound *until near the spot in the neck where strangulation had been effected;* and therefore (they add) we continue to think that Ch. Pichegru, ex-general, had committed suicide."

*Observations of M. Chaussier upon this report.*—In reading the account of this affair, says our author, one is struck particularly with the circumstance of the commissary of police pronouncing, by merely looking at the body, *that Pichegru had committed suicide the night before.* It appears that, by announcing this to the criminal tribunal, this opinion was already formed, and that the examiners *good-naturedly* followed the direction given to them, and adopted this pre-conceived notion with which they had been impressed. In the first report, the examiners give a detailed account of the features, &c. of the deceased, which was of no consequence to them, and omit entirely the only medical object of remark,—the state of the eyes and eyelids, the position and attitude in which the body was found: they only say it was *upon a bed;* but, whether dressed or undressed, or what was the state of the surrounding objects? They say they remarked a circular impression round the neck, and *afterwards* make mention of a black silk cravat, tied very tight. The point of the neck is not mentioned; nor do they decide whether the impression, which was most marked on the left side, was the effect of the change of colour of the folds of the skin; for it is to be observed, that a ligature drawn tight, and kept for some hours round a part of a dead body, forms a depression more or less deep, but does not change the colour. They then observe that the face was ecchymosed, and that this was the case over the whole body; but ecchymosis is a very different appearance from a livid, violet, or brownish colour, which is often observed after strangulation, or in some other cases; whereas, an ecchymosis consists of an extravasation and infiltration of blood in the tissue of a part. The examiners say that the feet were cold; but, at twelve hours after death, the trunk preserved, no doubt, some portion of warmth, though they do not say so. The blood was yet partly fluid, and the muscles of the neck and throat not quite stiffened. These circumstances should not have escaped the examiners, because, when a body is moved in that condition, the blood, by its weight, falls to the most dependent parts, and forms a species of over-distension *(engorgement)*.

In the second report, the remark upon the phlogosis of the stomach should have led them to examine, with the greatest care, the state of that viscus, the quantity and nature of its contents; into the regimen of the defunct, and the circumstances preceding his death. After taking out its contents, it should have been carefully examined, and slightly washed; since many substances, such as an infusion of the wild poppy, give the stomach a very remarkable reddish violet colour, as will many alimentary substances and some kinds of wine. The examiners then say that the *two lobes* of the lungs were gorged with blood: here there must be an error in the writer; and, lastly, they observe that the œsophagus was perfectly sound, to the spot in the neck where the strangulation had been effected, and yet they omit entirely to mention what kind of alteration it had undergone, or to what extent.

Our author next proposes two questions,—1st. Was the individual strangled? 2d. Did he strangle himself? The first question could only be answered by the examiners; and they have left it, as our learned author observes, very doubtful. With respect to the second question, M. Chaussier is evidently sceptical; and he notices the very strange expression used in the report, *on avait passé*, &c.; and he inquires, who had passed this stick? who had made this stick a tourniquet? Certainly, he continues, a firmly-resolved man may strangle himself by the method above named; but then the impression made by the cravat ought to be but slightly marked upon the neck, and still less so upon the œsophagus,—for the stoppage to the circulation in the veins of the neck produces a loss of recollection, and takes away the power of tightening the ligature; and, as the crime *might* have been committed by another, the examiners ought to have been content with saying that death was caused by strangulation.

We regret extremely that we must pass over a great mass of interesting matter. At page 322, and the following pages, is an interesting case of poisoning; and, at page 376, one still more so, relating to the body of a woman found hanging in her garden. But the length to which this article has already extended, obliges us to hasten to examine the third and last portion of the volume, which treats of *ecchymosis, sugillation, contusion,* and *bruise,* which, though apparently of little importance, become sometimes of material consequence, in relation to various points of medical jurisprudence.

M. Chaussier begins by defining the signification of these various terms. He appears to have a great partiality for etymology, and a sort of passion for tracing the origin and meaning of medical terms, as well as for restricting them to their precise meanings; and we think, as we before observed, that he carries this propensity so far as to raise a smile at the earnestness and solemnity of his manner. The essential character of ecchymosis is, according to our author, an effusion or extravasation of blood beneath the skin, and stands, therefore, contradistinguished from those livid spots which depend upon the stagnation and congestion of the blood in the capillary vessels, which are seen in the living body, sometimes surrounding the orbits in delicate, feeble, fatigued persons, and especially in many women at the time of menstruation. With respect to the word *sugillation,* which is adopted from the Latin, but which is not usually made use of in our language, we are told, after a great display of learning, and the quotation of authorities from Pliny to Van Swieten, that, in fact, it means neither more nor less than ecchymosis; though, if the Latin definition be adhered to, it properly means an extravasation of blood in consequence of a blow. Contusion implies a wound in the tissue of a part without a breach of surface; and in France the word *meurtrissure* (bruise) is used as equivalent to contusion: but, as precision of language is essential in all judicial inquiries, it is necessary to establish, says M. Chaussier, a difference in the sense and value of these two expressions. Contusion, he observes, is produced by an accidental fall, or a violent blow against some hard body, but without design; whereas the *meurtrissure* is a contusion produced by a combat between two or more persons, and therefore this word must not be used, unless it be actually proved that the blow was given by an adversary. He adds here, and we think with reason, "But this is, perhaps, giving too much consideration to mere words."

Among the numerous causes of ecchymosis, the medical man must recollect that the rupture of muscular fibres, a sudden movement in a direction contrary to the will of the party, or an effort of strength too long persisted in, may produce this appearance. A violent cough, or vomiting, or straining at stool, may do the same.

Some interesting examples of death from rupture of internal parts are given: one of a female, who, from travelling in a waggon, ruptured one of the veins of the right ovarium; a man, who had a considerable ecchymosis in the scrotum, in consequence of an effort in going to stool; a woman, who, in consequence of violent agitation and impatience whilst in labour, ruptured the iliacus internus muscle, and died. The following anecdote is directly in point:—Two men, who were at enmity with each other, coming in opposite directions, met in turning the corner of a street: one gave his antagonist a blow on the right side with his fist, and passed on; the other, surprised at this insult, turned suddenly round to run after his adversary, but almost immediately felt a violent pain in the calf of his left leg, which obliged him to stop and to go home, limping and suffering great pain. He made his complaint, and a judicial inquiry was commenced. Witnesses were called, and medical men were engaged to make a report of the case at two different times. In the first report, twenty-four hours after the rencontre, the examiner declared that there was no mark whatever of any blow received on the chest, but a tumefaction, or shining swelling, on the calf of the left leg, without any discolouration of the skin, and which appeared painful to the touch; and concluded that these appearances did not depend, as the complainant said, upon a blow received on that part, but were owing to the rupture of some fasciculi of muscular fibres at the time of his sudden turning round. At the time of the second report, eight days after the first, two medical men, who examined the leg, declared that there was neither swelling nor tension of the calf, but an ecchymosis, of a slight yellowish tinge; and in consequence of which they confirmed the judgment first given.

We shall not follow our author into the discussion of the distinctions of ecchymosis into traumatic, spasmodic, symptomatic, and spontaneous; they sufficiently explain themselves. The form, extent, and situation of an ecchymosis must also be considered; and upon these points the following illustration may not be unacceptable to our readers.—A young healthy woman complained that she had received a blow upon the left breast, eight days previously, and demanded to be examined, to prove the truth of her assertion. A physician and surgeon visited her, and found two superficial ecchymoses on the left breast, an inch apart from each other, without swelling or pain, one above the nipple, the other at the upper and inner part of the breast; each was of an elliptical form, six lines long and eight broad; their circumference was of a brownish red, without any diffusion or yellow tint around them. From all these circumstances, the examiners declared that these ecchymoses were not the result of a blow, nor were they received eight days previously; that they were probably produced by the mouth; and that on the right breast there were two superficial marks, diffused and yellowish, evidently the remains of an ecchymosis produced some days before. The result of the affair proved their opinion to have been correct.

Some pages are next devoted to the consideration of the distinction between ecchymosis and those livid marks often found upon the surface of different parts of the dead body; but we cannot enter into the discussion.

There is one more subject which we wish to notice, although we have scarcely room to do justice to it. Our author proposes this question, "Is it possible to determine whether wounds that are found upon an individual have been made by himself, or by an adversary?" Two cases bearing upon this question are related from Paré's works; in one of which an Englishman was robbed, had his throat cut, and was stabbed in several places, in the neighbourhood of Vincennes: he, however, made his way to the house of a peasant, and was taken to Paris, where his wounds were dressed, and he was enabled to relate the circumstances of his assassination. In the second instance, a German cut his throat, and gave himself several wounds in the thorax and belly, some penetrating the cavity, others superficial. It was believed that his servant was the assassin, because he slept in his chamber; he was arrested, and thrown into prison: but the wounded man, being visited by Paré, and the wound in his throat brought together, was enabled to speak, and to attest the innocence of the accused. In these cases, therefore, the intelligence, address, and attention of the surgeon are of the first importance; and our author gives excellent advice as to the conduct of the medical attendant upon such occasions, and dwells particularly on the necessity of paying attention to the antecedent and accompanying circumstances of the case.

He next criticises a report taken from the work of Devaux,—a case of a gun-shot wound of the hand, in which it was desired to ascertain whether it arose from the bursting of the gun in the hand of the wounded man, or in consequence of a shot fired purposely at him. We do not consider the report itself, nor the remarks upon it, as very conclusive; and therefore we proceed to the remaining section of the work, consisting of an article furnished by Baron Larrey, relative to a visit made by order of the commander-in-chief of the army in Germany, in the year 1813, to inquire into the cause and nature of the wounds of a considerable number of soldiers, wounded at the battles of Bautzen and Wurtchen. It appears that Bonaparte expressed great surprise at the number of men wounded in those actions, and some general officers suggested to him that a great number, especially of those disabled in the hands, or having the fingers torn or carried away, had wounded themselves on purpose to escape military service. At first he rejected this insinuation; but, being persuaded by several celebrated medical men, who declared they could easily distinguish a wound made voluntarily from one inflicted by the enemy, a jury, composed of the surgeon-in-chief and four principal surgeons of the army, was ordered to assemble, and visit all the soldiers wounded in the hand, or who had lost a finger, and to point out those who should have been found to have wounded themselves. So certain were they of the opinion that the jury would give, that it was already determined to select four from each corps of the army, (twelve in number,) to be shot at the head of their respective corps; but M. Larrey, before he complied with this order, endeavoured to convince the chief of the army of the particular causes that had, in this instance, produced this description of wound; which considerations, although highly rational, are too long to be extracted, and do not appear to have had the effect intended: and, therefore, 3500 soldiers, wounded in the hand or fingers, were assembled and examined in the most precise manner, the inquiry commencing at five o'clock in the morning of each day, and lasting four days. The result produced the following report:—1st. That almost all the wounds were produced by projectiles from fire-arms, and a small number by cutting weapons *(armes blanches)*. 2d. That the majority of those so wounded had also wounds in different parts of their bodies, or rents in their clothes, caused by the passage of balls. 3d. That the small number of wounded in which the above circumstances were not so evident, was composed principally of old soldiers, whose conduct could not be doubted; and, finally, the jury declare that there are no certain signs by which a difference can be perceived between a wound given voluntarily and one received involuntarily. This report was finally approved of, and the soldiers were sent back to their respective corps.

We have now brought this long article to a conclusion; and, although we are sensible of its many defects, we have, we conceive, extracted a sufficient portion of it to convince our readers that it is a work of real and intrinsic merit. We shall, as we before said, have occasion very soon to revert to the topic of medical jurisprudence, and shall then have an opportunity of doing further justice to the subject.

